# Construct–construct “rail technique” decreases screw strain during spinal deformity corrective maneuvers across a thoracic vertebral column resection: a cadaveric analysis

**DOI:** 10.1007/s43390-025-01195-9

**Published:** 2025-10-07

**Authors:** Alekos A. Theologis, Jason DePhillips, Izabella T. Lachcik, Jonathan M. Mahoney, Brandon S. Bucklen

**Affiliations:** 1 Department of Orthopaedic Surgery, University of California - San Francisco (UCSF), 500 Parnassus Ave, MUW 3Rd Floor, San Francisco, CA 94143 USA; 2 Scientific Affairs, Globus Medical Inc, Audubon, PA USA

**Keywords:** Spinal deformity, Correction techniques, Cantilever bending, Segmental compression, 3-column osteotomy, Vertebral column resection

## Abstract

**Purpose:**

To biomechanically compare screw strains above and below a vertebral column resection (VCR) during segmental compression (SC) and cantilever bending (CB) performed via traditional methods and a novel, construct-to-construct accessory rod (“rail”) technique.

**Methods:**

Eight cadaveric torsos underwent a VCR with 25^0^ kyphosis at T8 with pedicle screws implanted three levels above and below the VCR (T5-7; T9-11). Four screws (T6, T7, T9, T10) were instrumented with strain gauges to capture screw strains during SC and CB. Both deformity corrective maneuvers were performed over a traditional construct (central rod) and over a construct-to-construct accessory (“rail”) rod. Real-time screw strains were collected and peak strains were compared between corrective techniques.

**Results:**

Strains in screws closest to the VCR were significantly less during “rail” compression compared to traditional SC (T7: *p* = 0.015). Maximum screw strains were significantly lower during “rail” SC and CB compared to traditional SC (T6: *p* = 0.037; T7: *p* = 0.015) and CB (T6: *p* = 0.018; T9: *p* < 0.001). Total screw strain was more evenly distributed over all screws during “rail” compression and CB compared to traditional techniques, which concentrated strain at individual screws adjacent to the VCR.

**Conclusions:**

Performing segmental compression and cantilever bending across a lateral accessory construct-to-construct (“rail”) rod resulted in significantly lower strain on individual pedicle screws adjacent to a thoracic VCR compared to traditional SC and CB. As such, the “rail” may lessen risk of screw pull-out and screw plough during maneuvers to correct spinal deformities across a VCR.

## Introduction

Vertebral column resections (VCR) in the thoracic spine are the work-horse surgical technique to address disorders that afflict the anterior column of the spine (i.e. tumors and infections) and to correct rigid, complex thoracolumbar spinal deformities [1–5]. These procedures are surgically complex and carry relatively high rates of neurological complications, often associated with instrumentation, decompression, and spinal realignment during shortening and closure across the VCR site [6–8].

Two primary techniques used to shorten across a VCR are segmental compression (SC) and cantilever bending (CB). SC involves applying compression to pedicle screws adjacent to the VCR site, while CB entails positioning an under-contoured rod in the sagittal plane, securing it cranially or caudally to the VCR, pushing the rod down across the VCR, and securing it cranially or caudally to the VCR. Cantilevering shortens the posterior column and lengthens the anterior column through the VCR site [9]. Both techniques demand careful control to balance the extent of deformity correction with the forces exerted on the bone-screw interfaces. The forces required for correction can be substantial, leading to risks such as screw loosening, screw pull-out, and screw plough through the pedicle(s), which can impede the magnitude of correction and may cause construct failure or neural injury. Reported complication rates vary between 2–15%, with higher incidences seen in patients with compromised bone quality [10–13].

To address these challenges and reduce stress at the bone-screw interface during deformity correction across a thoracic VCR, a construct-to-construct strategy known as the “rail technique” was introduced by Theologis and Gupta in 2020 [14, 15]. This technique uses laterally positioned accessory rods, or "rails," that span a VCR, enabling SC and CB corrections to be applied through these rails. This method facilitates en-bloc movement of cranial and caudal spinal segments across the VCR, offering several advantages for safety and deformity correction, including (1) controlled compression across the VCR site, reducing translation risk; (2) asymmetric segmental compression and distraction through the VCR site, aiding in the correction of combined sagittal and coronal deformities; and (3) minimization of forces on the bone-screw interfaces around the VCR site, lowering the risk of peri-VCR screw failure [16–19].

While clinical and mechanical advantages of the "rail technique" have been documented [14, 15, 19], its biomechanical benefits relative to traditional SC and CB techniques in clinically relevant scenarios have yet to be directly assessed in a cadaveric setting. As such, this study aims to compare biomechanically the behavior of traditional SC to SC across the “rail” and traditional CB to CB using the “rail” technique in a cadaveric thoracic VCR model.

## Materials and methods

### Specimen preparation

Eight fresh-frozen cadaveric torsos (*n* = 8) were used for this study. Given the cadaveric nature of the entire study, neither institutional review board approval nor informed consent were required. Anteroposterior and lateral radiographs were taken before testing to screen cadaveric specimens for gross pathology. The medical history of each donor was reviewed. Presence of prior trauma, malignancy, deformity, and/or fractures were used as an exclusion criteria, as they were deemed to jeopardize testing. Prior to testing, specimens were thawed to room temperature. To evaluate the bone mineral density (BMD) (g/cm^2^), dual-energy X-ray absorptiometry (DEXA) scans were obtained using a Lunar Prodigy Scanner 8743, (GE Medical Systems, Waukesha, WI, USA) and a water-bath protocol [20].

### Surgical constructs

**Fig. 1:**
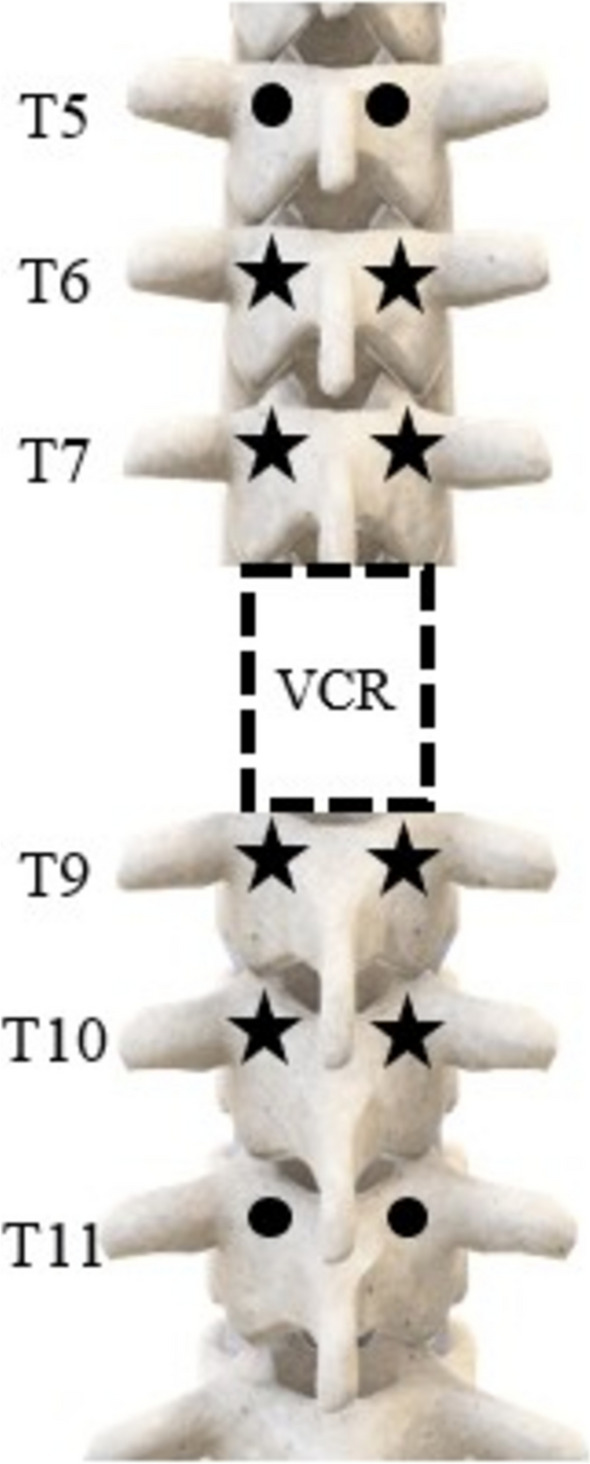
Schematic of the construct used in this study with pedicle screws placed three levels above and below the T8 vertebral column resection (VCR). The pedicle screws 2 levels above (T6, T7) and 2 levels below (T9, T10) the T8 VCR were instrumented with strain gauges

For all specimens, pedicle screws (CREO® 5.5, Globus Medical Inc., Audubon, PA) with diameters of 5.5 mm or 6.5 mm and lengths of 40 mm or 45 mm (sizes based on specimen-specific anatomy) were inserted bilaterally from T5 to T11, excluding T8. Pedicle screws two levels above (T6, T7) and two levels below (T9, T10) the planned VCR site (T8) were instrumented with linear strain gauges (KFH-03–350-C1-11L3M3R, Omega Engineering, Inc., Stamford, CT) (Fig. 1) [19]. Gauges were mounted lengthwise, parallel to the axis of the screw, in order to optimize strain measurements [19]. Once mounted, each gauge was layered with a coating of metal epoxy (DEVCON® Plastic Steel® Liquid (B), ITW Performance Polymers, Danvers, MA) to protect the mechanical and electrical integrity of the gauge [19]. All thoracic pedicle screws were inserted using the approach described previously by Kim and Lenke [21] following a lateral-to-medial trajectory parallel to the superior endplate until approximately 60–75% of the vertebral body was penetrated, as seen in lateral radiographs.

**Fig. 2:**
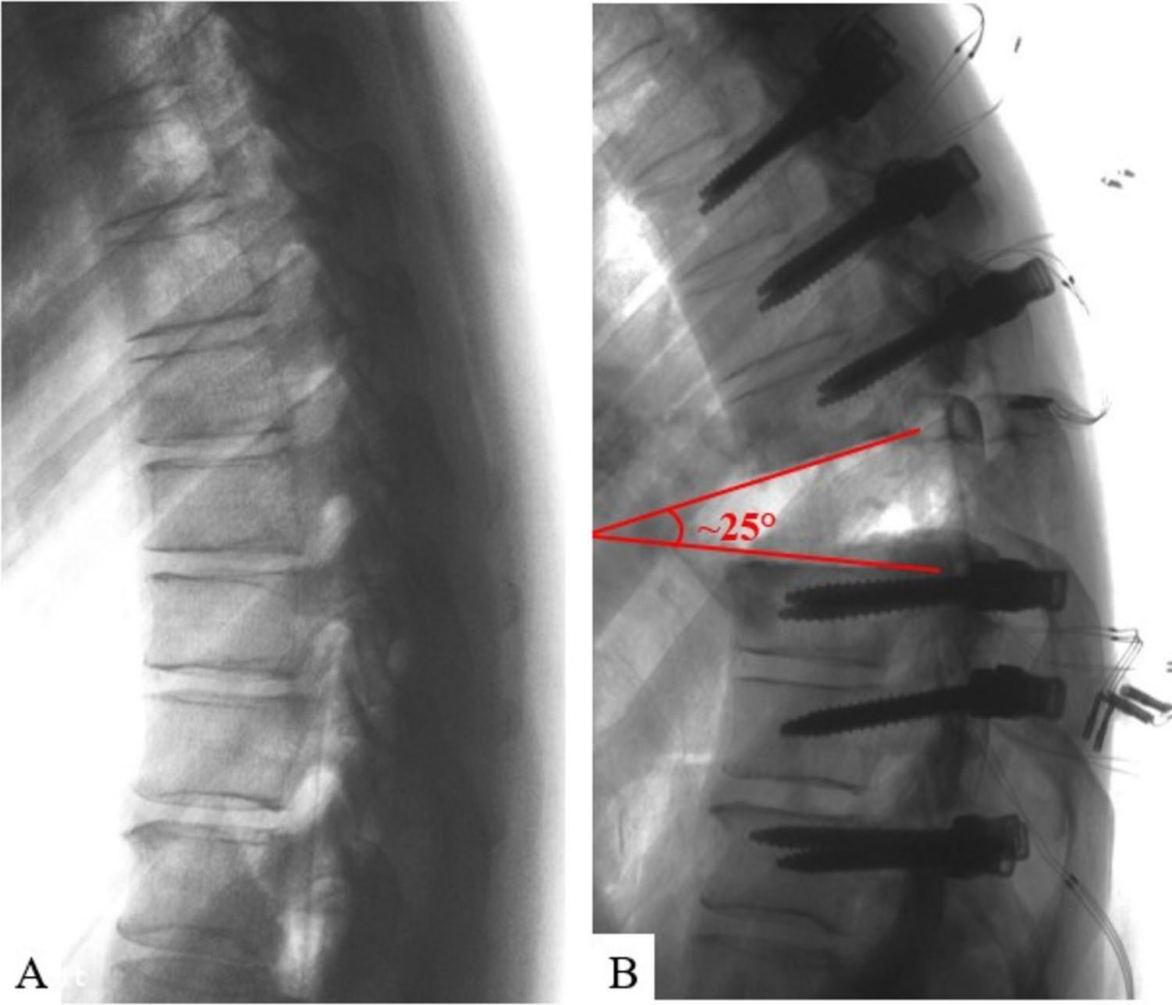
Lateral radiographs of **A** intact specimen and **B** 25° kyphotic deformity induced through the T8 VCR

Once screws were inserted, a posterior VCR was performed at T8 following standard, step-wise resection of appropriate soft-tissues and osseous anatomy [22]. Resection of the posterior elements, including the entire lamina of T8, approximately 75% of the caudal aspect of the T7 lamina, approximately 50% of the cranial aspect of the T9 lamina, and the ligament flavum at T7-8 and T8-9, were removed. This was followed by the removal of the T8 pedicles as well as approximately 3 cm of the T8 rib heads bilaterally. The entire T8 vertebral body, including its entire posterior wall, as well as the posterior longitudinal ligaments at T7-8 and T8-9 were removed. At this point, a 25° kyphotic deformity through the T8 VCR site (inferior endplate of T7 to the superior endplate of T9) was introduced and confirmed to simulate common clinical scenarios (i.e. kyphosis from a tumor, infection, congenital anomaly, healed fracture) (Fig. 2). Based on the planned deformity correction technique (standard vs. “rail”), the respective construct (details below) was placed in situ on the left and right sides from T5 to T11 to hold this kyphotic deformity in position and provide provisional stability across the VCR site. A titanium corpectomy spacer (FORTIFY®, Globus Medical Inc.) was then properly sized (12 mm core; 14 × 16 mm endplates; superior endplate 0^0^ lordosis; inferior endplate 0^0^ lordosis; heights varied by height of corpectomy defect from T7 inferior endplate to T9 superior endplate), inserted, and expanded in place of the T8 vertebral body.

All specimens then underwent deformity correction with SC being performed on the left side followed by CB on the right side. Deformity correction was applied using both techniques: traditional technique (central rod) and construct-to-construct technique (“rail” rod). The order of which technique (standard vs. “rail”) was applied first was randomized to minimize any effect of implant fatigue on the results of the study.

To ensure equivalent deformity correction across techniques, standardized procedures were used for both segmental compression and cantilever bending. For segmental compression, a parallel compressor was used with five notches applied uniformly across all specimens. For cantilever bending, under-contoured rods were pre-bent to an identical sagittal profile using a reference rod as a bending template. In both cases, fluoroscopic imaging was used to confirm equivalent angular correction.

### Deformity correction

#### Traditional segmental compression

**Fig. 3:**
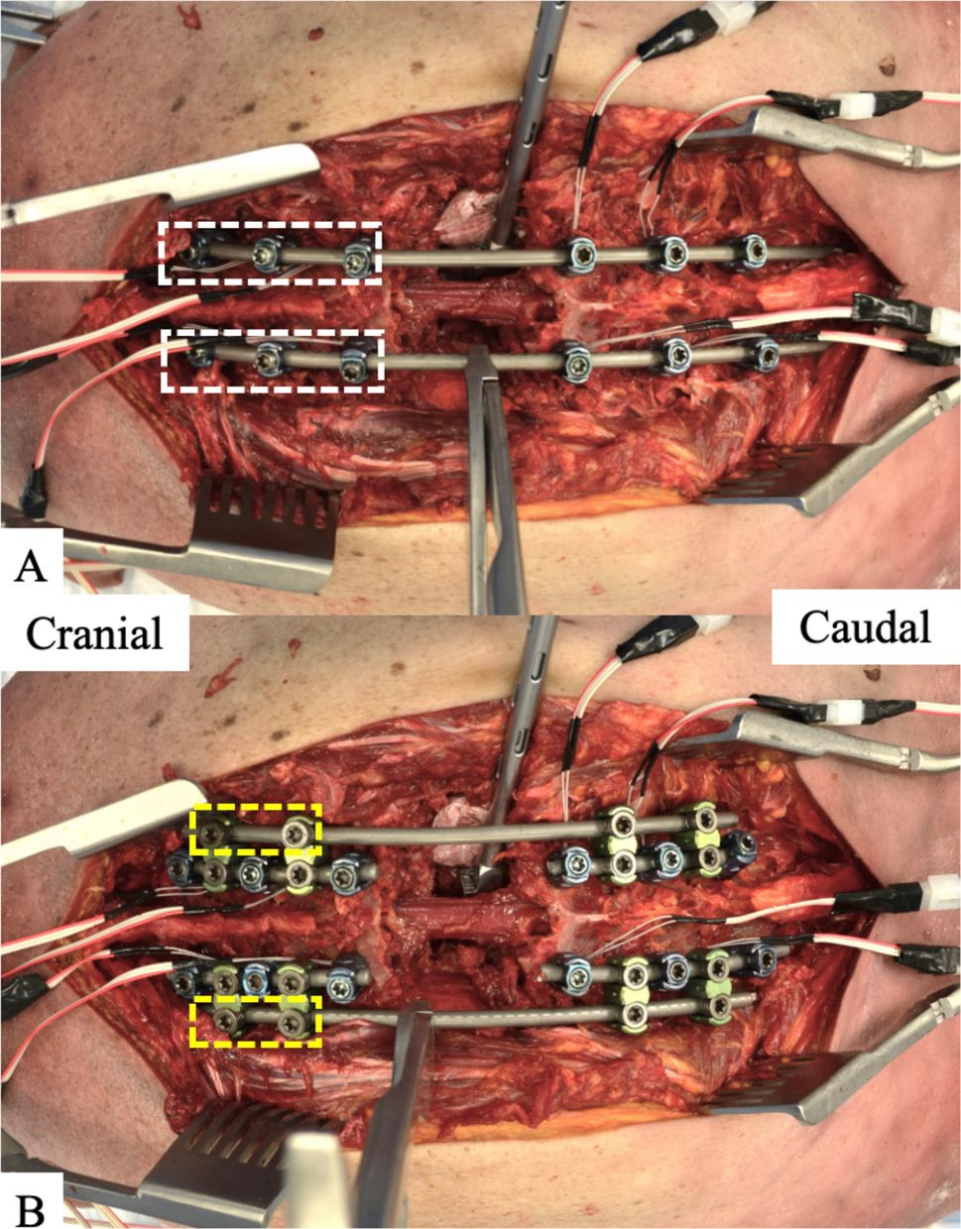
Images of **A** traditional segmental compression (SC) in which the cranial screws’ set screws (white boxes) remain loose during compression across the osteotomy site, and **B** compression across the “rail” in which only the set screws on the accessory/ “rail” rod of the cranial W-connectors (yellow boxes) remain loose during compression across the osteotomy site

A 5.5 mm cobalt chrome rod was placed bilaterally spanning the VCR from T5-11. All locking caps caudal to the VCR bilaterally were tightened while all locking caps cranial to the VCR bilaterally remained loose (Fig. 3A). A rod holder was then secured to the left primary rod and a parallel compressor was engaged with the rod holder and the left T7 screw. The parallel compressor was used to apply 5-notches worth of compression across the T8 VCR site.

#### Traditional cantilever bending

**Fig. 4:**
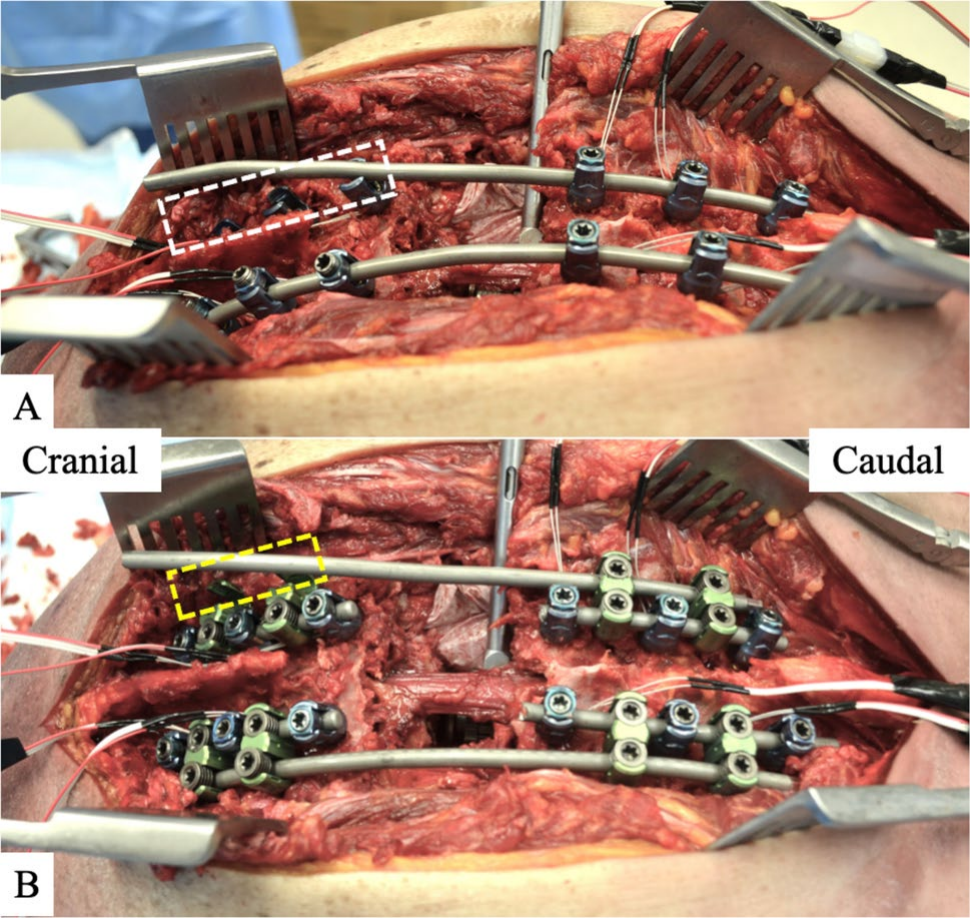
Images of **A** traditional cantilever bending (CB) and **B** CB using the “rail” rod. For traditional CB (**A**), the rod was secured to all pedicle screws caudal to the osteotomy site and then its cranial portion was pushed down into the screws cranial to the osteotomy (white box). For CB over the “rail” (**B**), the lateral accessory rod was secured to the two W connectors caudal to the osteotomy site and then its cranial portion was pushed down into the W connectors cranial to the osteotomy site (yellow box)

The right-side rod was then removed and replaced with an under-contoured 5.5.mm cobalt-chrome rod using the following technique (Fig. 4A). This under-contoured rod was then secured to the screws caudal to the VCR site and the set screws were finally tightened. The set screws cranial to the VCR site on the left side were loosened to facilitate additional correction through the VCR site during CB. This was then followed by a cantilever maneuver from caudal to cranially in which the cranial aspect of the rod was pushed down into and secured in the two supra-adjacent pedicle screws above the VCR site (T6, T7).

#### “Rail” compression

Four short 5.5 mm cobalt chrome rods were first placed and secured bilaterally into screws from T5-7 and from T9-11 (Fig. 3B). W-connectors were then connected to these rods bilaterally at T5-6, T6-7, T9-10, and T10-11. Two 5.5 mm cobalt chrome rods were then placed laterally into these W connectors bilaterally, thus creating the “rails” (Fig. 3B). The set screws were finally tightened on the four short midline rods and on the W-connectors caudal to the VCR. Set screws on the W-connectors cranial to the VCR remained loose. A rod holder was then secured to the lateral accessory/ “rail” rod on the left side and 5-notches worth of compression were applied across the rod holder and the W-connector at T6-7.

#### “Rail” cantilever bending

The right-sided lateral accessory rod was then removed and replaced with an under-contoured 5.5 mm cobalt chrome rod using the following technique (Fig. 4B). The under-contoured rod was first secured laterally to the W connectors caudal to the VCR site and these set screws were finally tightened. The set screws on the W connectors’ lateral rod cranial to the VCR site on the left side were then loosened to facilitate additional correction through the VCR site during CB. This was then followed by a cantilever maneuver from caudal to cranial in which the cranial aspect of the rod was pushed down into and secured laterally in the two W connectors above the VCR site.

### Statistical analysis

Statistical analyses were performed using IBM SPSS® Statistics (SPSS® v22, IBM Corp., Armonk, NY, USA). A simple linear regression analysis was run to analyze the relationship between BMD and screw strain. All specimens underwent both traditional and “rail” deformity correction techniques in a randomized order to minimize sequencing bias. Accordingly, statistical comparisons were performed using paired t-tests, with each specimen serving as its own control (*n* = 8 matched pairs). This within-subject approach increased statistical power and reduced variability attributable to anatomical differences between specimens. For all tests, a statistically significant difference was defined as *p* < 0.05. A post-hoc power analysis was performed using G-Power (Version 3.1.9.7) based on the observed effect size at T7 during bending (effect size = 1.04). With a sample size of 8 dependent pairs, *α* = 0.05, and a two-tailed test, the calculated power was 0.82. This level of statistical power is considered reasonable given the logistical and anatomical constraints inherent to cadaveric biomechanical studies.

## Results

Demographic information for the 8 cadaveric specimens (4 male, 4 female) used in this study are presented in Table 1. The average age was 58.5 ± 9.4 years (range, 51–75 years), and the average BMD was 1.16 ± 0.22 g/cm2. Screw strain was not significantly associated with specimen BMD (*p* = 0.918).

**Table 1:** Summary of demographics for cadaveric specimens

Specimen	Sex	Age (years)	BMD (g/cm^2^)	T-score	1st technique
1	M	61	1.169	− 0.5	Rail
2	F	75	0.671	− 4.2	Rail
3	M	57	1.059	− 1.4	Rail
4	M	51	1.213	− 0.2	Rail
5	F	54	1.183	− 0.1	Traditional
6	M	58	1.480	2.0	Traditional
7	F	69	1.224	0.2	Traditional
8	F	43	1.302	0.9	Traditional

^*^BMD Bone mineral density, *M* Male, *F* Female

### Compression

**Fig. 5:**
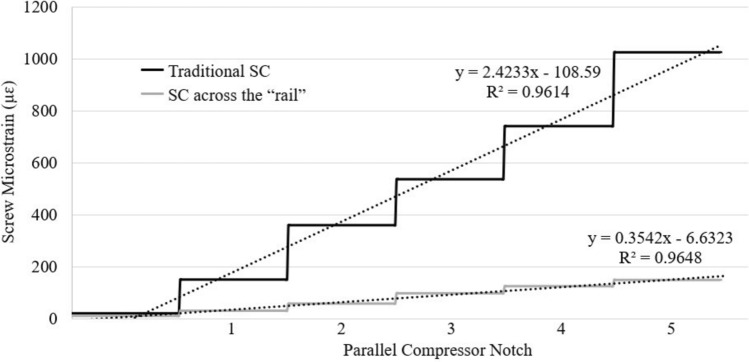
Rate of increase in strain at the worst-case screws for each compression technique: T7 during traditional segmental compression (SC) (black line) and T9 during “rail” SC (grey line) using the notches of the parallel compressor

**Fig. 6:**
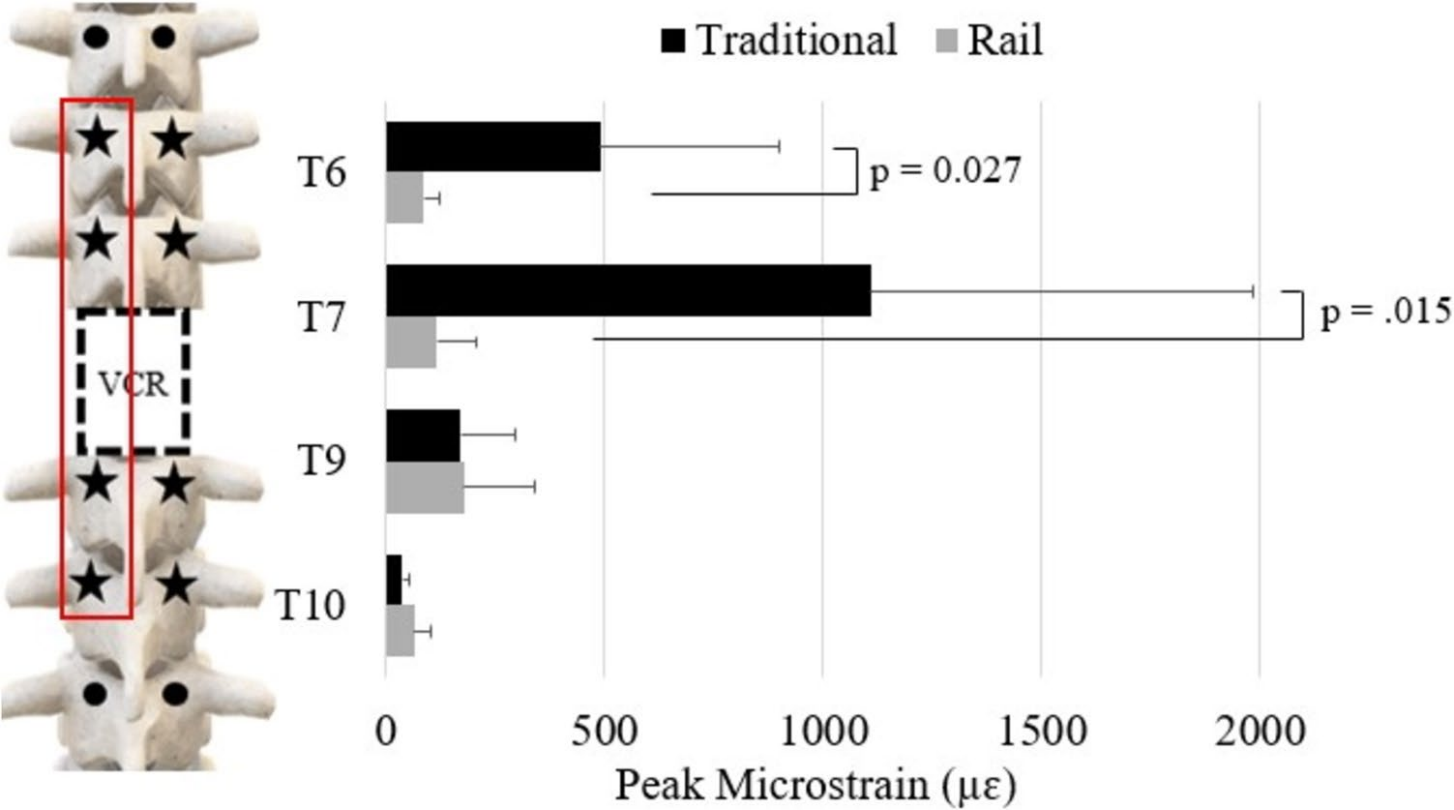
Peak strains for each screw on the left during traditional segmental compression (SC; black bars) and “rail” compression (grey bars) using a maximum grip on the parallel compressor

For compression, maximum screw strains occurred at T7 for traditional SC (T7: 1108 ± 876 µɛ) and at T9 for “rail” compression (178.8 ± 162 µɛ). For traditional SC, the rate of strain increase at T7 was 584% higher compared to the rate of strain increase at T9 during “rail” compression (Fig. 5). Screw strains at T6 and T7 were also significantly greater during traditional SC than “rail” compression (T6: 491.1 ± 408.8 µɛ vs. 87.4 ± 33.8 µɛ, *p* = 0.031; T7: 1108 ± 876 µɛ vs. 115.3 ± 92.0 µɛ, *p* = 0.015) (Fig. 6). At the respective location of maximum screw strain, “rail” compression had an 84% lower strain than traditional SC. Additionally, screw strain was more evenly distributed across all four instrumented screws during “rail” compression (T6—20%, T7—26%, T9—40%, T10—14%) compared to traditional SC in which the screw strain was most focused on the peri-VCR screw (T7—61% *v.* T6—27%, T9—9%, T10—2%).

### Cantilever bending

**Fig. 7:**
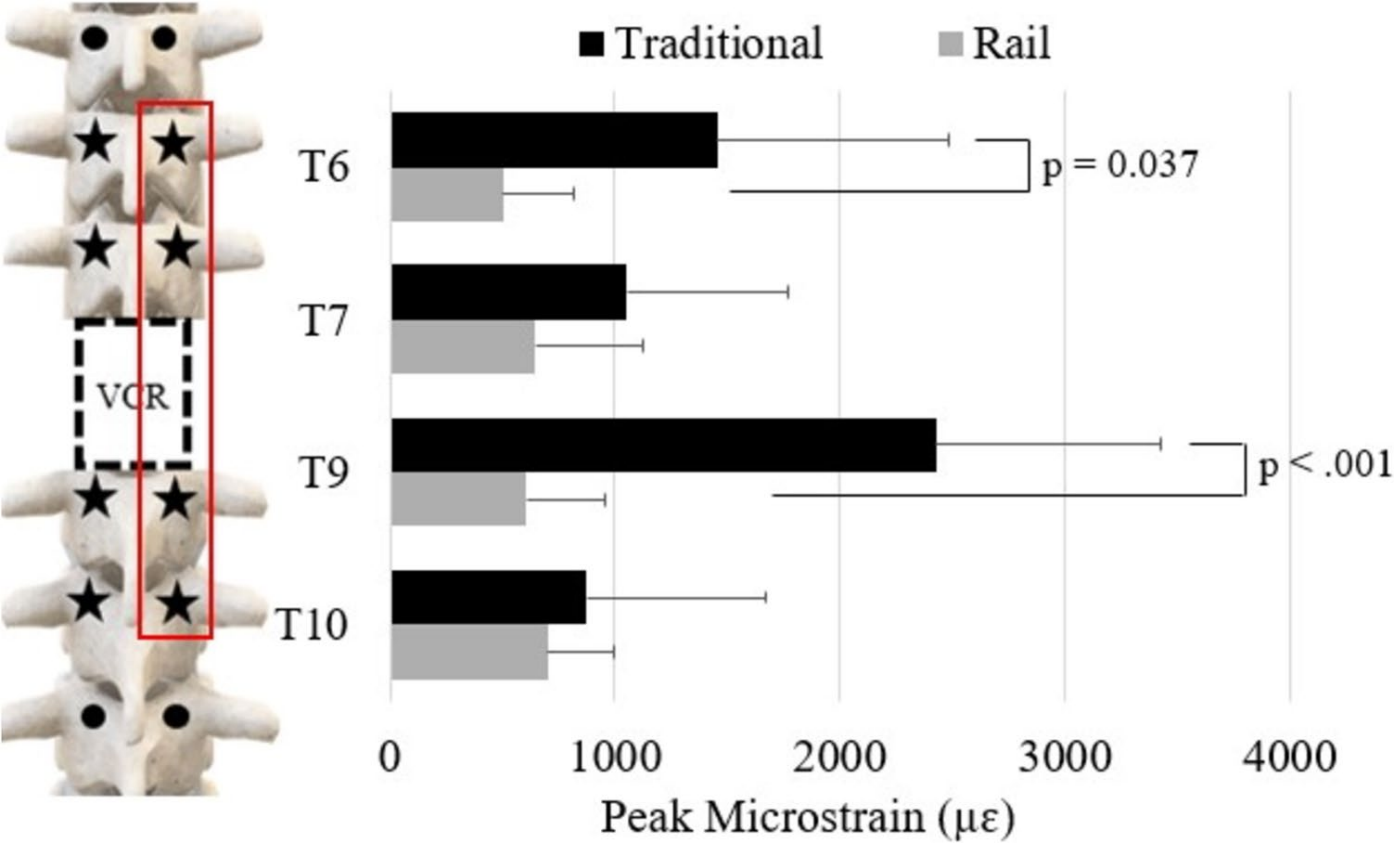
Peak strain of each screw on the right during traditional cantilever bending (CB; black bars) and “rail” CB (grey bars)

For CB, maximum screw strains occurred at T9 in traditional CB (2425 ± 1005 µɛ) and T10 in “rail” CB (703.5 ± 288.1 µɛ). The screw strains at T6 and T9 were significantly greater during traditional CB than “rail” CB (T6: 1454 ± 1031 µɛ vs. 500.4 ± 320.7 µɛ, *p* = 0.019; T9: 2425 ± 1005 µɛ vs. 606.8 ± 350.3 µɛ, *p* < 0.001) (Fig. 7). At the respective locations of maximum screw strain, “rail” CB resulted in 71% less strain than traditional CB. Additionally, screw strain was more evenly distributed across all screws during “rail” CB (T6—22%, T7—26%, T9—25%, T10—29%) compared to traditional CB (T6—25%, T7—18%, T9—42%, T10—15%).

**Fig. 8:**
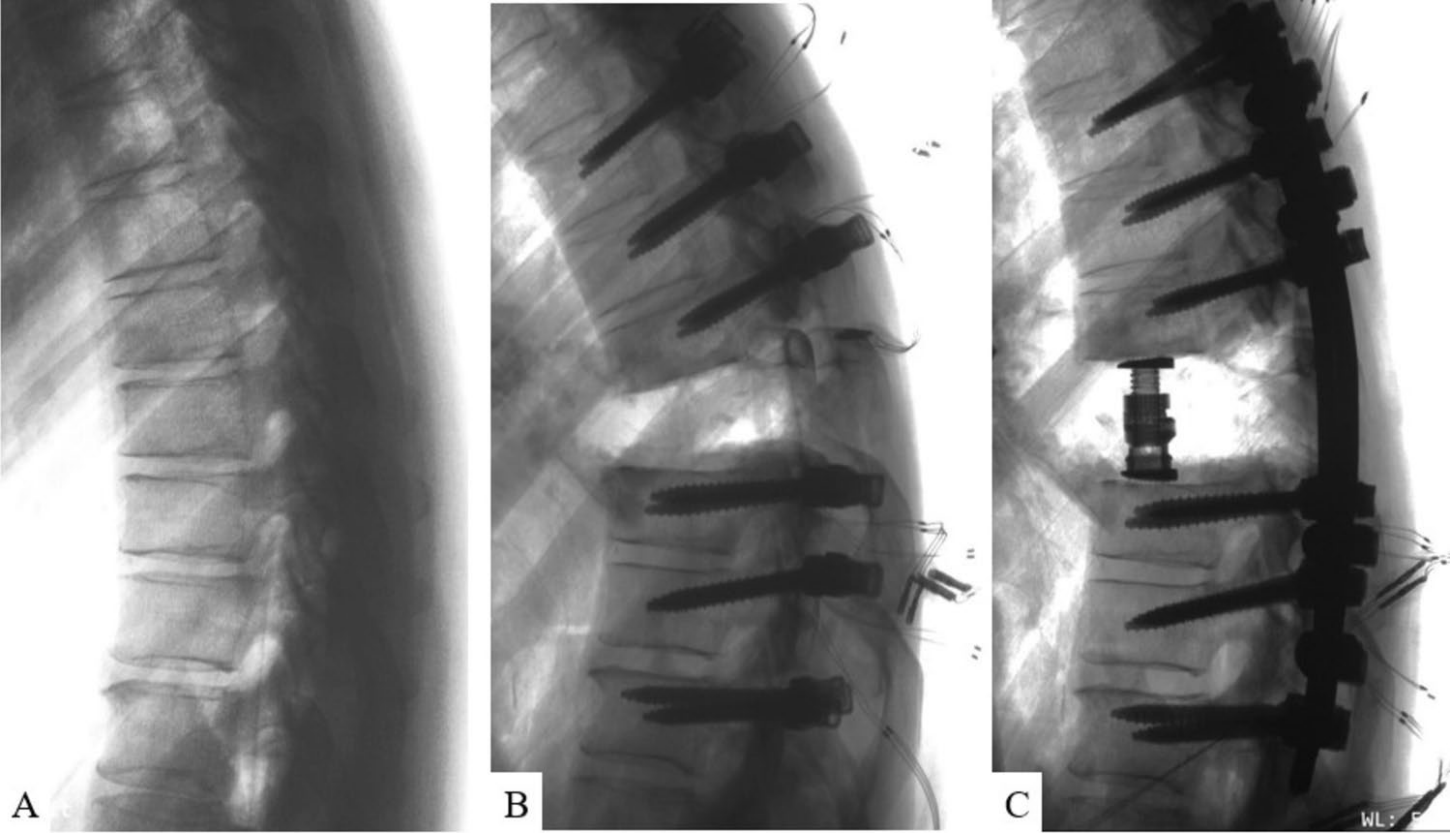
Lateral radiographs of **A** intact specimen, **B** T8 vertebral column resection (VCR) with a 25^0^ kyphotic deformity, and **C** realigned spine through the VCR site with T8 anterior column reconstruction using an expandable titanium cage

Both surgical strategies (traditional and “rail” compression and CB) effectively allowed for restoration of appropriate spinal alignment in all cadavers (Fig. 8). There were no known instances of screw pullout or screw plough in either technique.

## Discussion

This study aimed to evaluate screw strains during traditional spinal deformity correction techniques (SC and CB) across a thoracic VCR site and compare them to the same techniques performed using a construct-to-construct accessory rod ("rail") in a cadaveric environment. During correction of a 25° kyphotic deformity through T8, this study demonstrates the following 3 primary outcomes: (1) screw strains closest to the VCR were significantly lower when using the "rail" with SC and CB compared to traditional SC and CB; (2) peak screw strains were reduced by 84% during "rail" compression and by 71% during "rail" CB compared to their traditional counterparts; and (3) strain distribution across screws was more evenly distributed during "rail" compression and "rail" CB, while traditional techniques concentrated strain on screws adjacent to the VCR site. Collectively, these findings provide important biomechanical insights into this emerging surgical approach, which has been primarily supported by clinical case reports highlighting its potential to correct complex spinal deformities safely, as well as a recent mechanical investigation.

The aforementioned biomechanical results can be attributed to the “rail” rod effectively allowing en-bloc shortening across a VCR in which multiple spinal units/segments above and below the VCR move together rather than segmental correction in which the required corrective forces are primarily concentrated on individual screws, particularly the screws adjacent to an osteotomy site. These sentiments are supported by a recent mechanical analysis from *Theologis *et al*.* using PCF foam blocks in which it was found that strains in screws closest to a simulated osteotomy were significantly less during “rail” compression (288.6 ± 162.2 µɛ) compared to traditional SC (2184 ± 645.7 µɛ) as well as during “rail” CB (762 ± 346.1 µɛ) compared to traditional CB (1817 ± 327.1 µɛ) [19]. Additionally, total screw strains were more evenly distributed across screws during “rail” compression and “rail” CB (each screw < 20% strain) compared to traditional maneuvers in which the majority of the strain (30–75%) was felt by screws adjacent to the simulated osteotomy site [19]. While 10PCF foam provides a controlled mechanical environment that allows for reproducible and standardized conditions, enabling proof-of-concept testing for novel testing technologies, they do not fully replicate the heterogeneity and anisotropy of cadaveric or in vivo bone. Additionally, this prior mechanical investigation did not involve a resected component of the foam (i.e. there was no simulated VCR/corpectomy) across which shortening or angular deformities were corrected. As such, a more clinically relevant scenario using a cadaveric specimen that involved a true VCR with an associated kyphotic deformity and anterior column reconstruction with a cage was felt to be necessary to validate the prior mechanical findings. Importantly, the results of the current cadaveric analysis align closely with those of the prior mechanical investigation. By distributing necessary corrective force across multiple spinal units and minimizing strain placed on the screws adjacent to a VCR, the “rail technique” lessens the risk of screw pull-out during cantilever bending and screw plough through a pedicle during segmental compression. While this is important for all deformity corrections, it is most necessary in patients who have poor bone quality or require significant spinal realignment, as any loss of fixation in these settings may prevent safe or achievable deformity correction [23–26]. While no screws were noted to plough through the pedicle or pull out during traditional SC and CB maneuvers in this cadaveric testing model, each technique focuses relatively high forces on individual screws increases the risk of pedicle failure occurring in patients with compromised bone compared to the respective “rail” maneuvers. While the authors do not use strict criteria based on age, bone quality, and/or deformity severity to implement the “rail” technique, it is felt that patients with more advanced age, severely compromised bone quality, and greater magnitude deformities are particularly good candidates for use of the “rail” technique. Given the findings of this study and aforementioned mechanical benefits, the authors of this study use the “rail technique” for all thoracic corpectomies/3-column osteotomies to correct focal deformities secondary to tumor, infection, fractures, and proximal/distal junctional pathology.

Clinical utility and safety of the “rail technique” have been demonstrated in two prior case reports. In its first reported use, *Theologis *et al*.* presented a case of a 65-year-old female with osteoporosis who underwent an uncomplicated revision C2-T10 posterior spinal instrumented fusion (PSIF) with shortening across a T4 VCR to correct a junctional failure/kyphosis at T4 above a prior T5-pelvis PSIF [14]*.* In a separate case report with an accompanying step-by-step surgical video, *Collins *et al*.* highlighted the efficacy of the “rail technique” to correct a severe proximal junctional fracture/kyphosis across a T9 VCR above a prior T10-pelvis posterior instrumented fusion in a 69-year-old female [15]. While both of these two prior reports focused on the use of the “rail technique” to correct proximal junctional fractures and kyphosis, the “rail technique” (as described in this biomechanical analysis) can be used to stabilize and correct focal malignment of the spine from any pathological process that afflicts the anterior spinal column. For example, the first author of this study has used the “rail technique” numerous times and as a primary method of deformity correction across a thoracic VCR to address kyphosis/kyphoscoliosis from acute or chronic vertebral body fractures (compression or burst), single-level or multi-level pathological fractures from metastatic lesions and vertebral osteomyelitis/osteodisciitis, primary spine tumor en-bloc resections, and complex, multiplanar congenital anomalies (i.e. hemivertebrae, block vertebrae). That the purported and observed mechanical and safety benefits afforded by the “rail technique” during surgery are corroborated by this study’s biomechanical data is a testament to the technique’s integrity, practicality, and value.

The findings of this study should be considered in the context of its limitations. Due to the cadaveric nature of this study, the biomechanical results are limited in that they do not replicate the in vivo condition in which a variety of muscular interactions create forces across the motion segments of the spine [27]. While contractions and forces from the paraspinal muscles and intercostal musculature during surgery may influence the results of the individual surgical strategies, their potential influence is considered less relevant in this study given the results presented are from comparative analyses between 2 surgical strategies performed in a within-subject, paired design, in which each specimen served as its own control, which allowed for control of inter-specimen variability. Another limitation is that strain was measured on the screw shaft rather than directly at the bone-screw interface. As such, this study assumes that high screw strain correlates with high interfacial strain. While this provides a reasonable surrogate for relative comparisons, it may not fully capture complex loading conditions such as off-axis or torsional forces. Future advancements in testing modalities may improve the ability to more directly assess bone-screw interface mechanics and refine the relationship between screw strain and fixation stability. Since there were no screw-bone interface failures, this is a study of force distribution and construct mechanics, not cadavers or bone density consideration. A clinically useful analysis for future investigations would be a correlation of Hounsfield units to forces applied and screw pull out. Another limitation is that the recorded strain magnitudes in this study are a function of the instrumented screws’ lengths, diameters, and material properties, such as Modulus of Elasticity. While it can be assumed that the magnitudes of strain will be affected by changes in these three factors, the elucidated mechanical phenomenon/pattern seen in this study is likely be seen across all screw configurations. Despite these limitations, the results of this study are unique and lend important biomechanical credence to the purported surgical efficacy and safety profile of the “rail technique” reported in prior clinical reports. As such, the results of this study may be considered an important addition to the literature on surgical strategies to correct complex spinal deformities of the thoracolumbar spine.

## Conclusions

In this cadaveric analysis, performing compression and cantilever bending across an accessory rod (“rail”) that connects to instrumentation above/below a thoracic VCR resulted in significantly lower strain on individual pedicle screws adjacent to the VCR site compared to traditional segmental compression and CB. The “rail technique” used in this study demonstrated the potential to lessen the risk of screw pull-out and screw plough during maneuvers to correct complex spinal deformities of the thoracic spine. This study’s biomechanical findings corroborate previously reported clinical advantages of the construct-to-construct “rail technique” for correction of complex thoracic spinal deformities.

## Data Availability

The data that support the findings of this study are available from the corresponding author, AAT, upon reasonable request.
